# Successful renal transplantation after recovery from acute disseminated encephalomyelitis in a child with end-stage renal disease

**DOI:** 10.4103/0972-5229.68228

**Published:** 2010

**Authors:** Guruprasad P. Bhosale, Veena R. Shah, Hargovind L. Trivedi

**Affiliations:** **From:** Department of Anesthesia and Critical Care, Smt. G. R. Doshi and Smt. K. M. Mehta Institute of Kidney Diseases and Research Centre & Dr. H. L. Trivedi Institute of Transplantation Sciences, Civil Hospital Campus, Asarwa, Ahmedabad, Gujarat – 380 016, India; 1 Department of Nephrology and Transplantation, Smt. G. R. Doshi and Smt. K. M. Mehta Institute of Kidney Diseases and Research Centre & Dr. H. L. Trivedi Institute of Transplantation Sciences, Civil Hospital Campus, Asarwa, Ahmedabad, Gujarat – 380 016, India

**Keywords:** Acute disseminated encephalomyelitis, end-stage renal disease

## Abstract

Acute disseminated encephalomyelitis (ADEM), seen mostly in children, is an acute demyelinating disease, affecting mainly the white matter of brain and spinal cord. We report an unusual case of ADEM in an 11-year old boy with endstage renal disease, who underwent hemopoietic stem cell transplantation prior to renal transplantation. He needed admission to the intensive care unit and required mechanical ventilation. He responded to intravenous injection of steroids and upon recovery, underwent renal transplantation successfully.

## Introduction

Acute disseminated encephalomyelitis (ADEM) represents an acute demyelinating disease affecting the central nervous system. It is an immune-mediated inflammatory disorder characterized by widespread demyelination that predominantly involves the white matter of the brain and spinal cord.[1] It is mostly seen in children. There have been reports of ADEM after renal transplantation.[2,3] We report an unusual case of ADEM in a child suffering from end-stage renal disease who underwent hemopoietic stem cell transplantation (HSCT) prior to renal transplantation. Later the child underwent successful renal transplantation after recovery from ADEM.

## Case Report

The patient was an 11-year old boy with end-stage renal disease due to obstructive uropathy. He was diagnosed to have posterior urethral valves at the age of two years and had undergone valve fulgration twice. This was followed by bilateral ureteric reimplantation for bilateral hydronephrosis at the age of seven years. At the age of 10, he developed end-stage renal disease and was put on maintenance hemodialysis through an arterio-venous fistula. He underwent bilateral native nephrectomies for the control of hypertension. He was being prepared to undergo renal transplantation, the donor being his mother. A week prior to the scheduled renal transplant, he underwent an allogeneic hemopoietic stem cell transplantation (HSCT) with mother as donor, as a part of tolerance induction protocol. The conditioning regime prior to stem cell transplantation included a single dose of anti-thymocyte globulin (ATG) 1.5 mg./kg and a single dose of cyclophosphamide 15 mg./kg.

**Figure 1:**
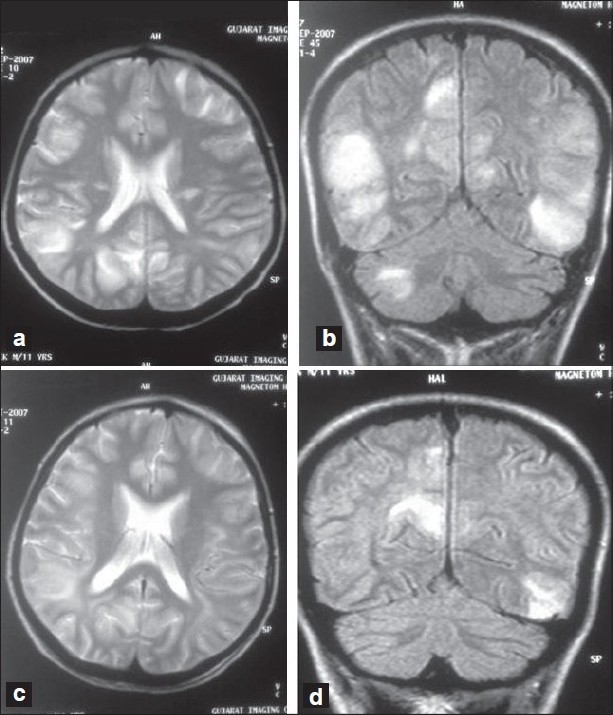
T2 weighted (a) and FLAIR (Fluid attenuated inversion recovery) MRI images (b) showing patchy areas of confluent hyperintensities involving cortical and subcortical, bilateral, frontal, posterior temporal, parietooccipital lobes and right cerebellum. MR, one week after steroid treatment, showing resolution of lesions in T2 weighted (c) and FLAIR- (d) images

Five days after HSCT, he developed fever, malaise, sore throat, dry cough followed by sudden change of behaviour and agitation. He was admitted to the intensive care unit. On presentation, his pulse was 110/min and BP was 180/120 mm Hg. Remaining CNS examination was normal. His BP was controlled with oral antihypertensives and Inj. Sodium nitroprusside infusion. Even after BP came under control, his abnormal behaviour continued. He became more and more drowsy, but there was no focal neurological deficit. On the same day, he developed a tonic-clonic seizure with difficulty in breathing. Seizure was controlled with phenytoin. For protection of airway, trachea was intubated and patient was put on ventilator on pressure support mode. CSF examination did not reveal any positive findings. In view of his rapidly deteriorating CNS condition, magnetic resonance imaging (MRI) brain was done which showed patchy areas of confluent hyperintensities involving cortical and subcortical, bilateral, frontal, posterior temporal, parieto-occipital lobes and right cerebellum on T2 W (weighted) and FLAIR (Fluid attenuated inversion recovery) images, consistent with the diagnosis of ADEM [Figure 1]. He was given Inj. Methyl prednisolone 500 mg intravenously over 1 hour for three consecutive days followed by oral corticosteroids in tapering doses. Patient improved dramatically and was weaned off the ventilator on third day. However, he still had difficulty in deglutition and aphasia, which gradually improved over a week after oromotor physiotherapy. Repeat MRI brain showed regression of the cerebral lesions and near complete resolution of right cerebellar lesion suggestive of improvement [Figure 1]. One week later, patient completely recovered with normal speech, swallowing and CNS function within two weeks.

In the following week, he underwent renal transplantation under combined general and epidural anesthesia. He received two-drug immunosuppression with steroid and mycophenolate mofetil. The graft functioned soon after transplantation and S. creatinine level was 2.3 mg./dl on 2^nd^ postoperative day. On postoperative day 21, he was discharged with normal renal function with S. Creatinine level of 1.3 mg.dl^-1^. At one year follow up, he is having no neurological sequelae and a good graft function with S. creatinine of 0.79 mg./dl.

## Discussion

ADEM, mostly seen in children, is an immune-mediated disorder, occurring typically after an exanthematous viral or bacterial infection or following vaccination.[4] Possibly, a T cell mediated autoimmune response to myelin basic protein, triggered by an infection or vaccination, underlies its pathogenesis.[5] The clinical picture is usually a viral prodrome followed by development of neurological signs and symptoms like paresthesia, paralysis and seizures. Sometimes the neurological condition may rapidly deteriorate to coma and death.[2] Neuroimaging is extremely important in establishing the diagnosis of ADEM.[1] MRI brain typically shows diffuse bilateral CNS demyelination with extensive involvement of white mater.[6]

ADEM has been shown to be associated with HSCT.[7,8] Woodard *et al*, reported two cases of ADEM after allogeneic HSCT in their analysis of 405 patients over a 10 year period and stressed that early and aggressive diagnostic measures, in combination with appropriate medical therapy and intense rehabilitation may improve prognosis in these pediatric patients.[7] Incidence of severe neurological complications after HSCT have been shown to be as high as 14% and the risk factors identified are allogeneic HSCT especially from an unrelated donor, the development of severe acute graft versus host disease (GVHD) grade>2 and the use of total body irradiation.[8] In our patient, the most probable cause for ADEM seems to be the allogeneic HSCT even though he received it from a related donor and none of the other risk factors were present.

Upper respiratory tract infection is seen in as many as 50% of cases before the onset of ADEM.[9] In our case, in view of fever, malaise, sore throat and dry cough, there could have been an upper respiratory tract infection, which might have triggered an autoimmune response. Furthermore, ATG has also been implicated in reactivation of viruses like Cytomegalovirus, Epstein-Barr Virus and Hepatitis B virus.[9–11] In our case, the immune-mediated response of ADEM could have been due to infusion of ATG as a part of the conditioning regime prior to HSCT. However, there has been no report of any association between ATG and ADEM in the literature. Furthermore, we could not isolate any bacterial or viral pathogen and the viral serological tests were also negative. In a recent study by Murthy *et al*, despite vigorous attempts to identify microbial pathogens in 18 patients, only one patient had Epstein-Barr virus isolated as the definite microbial cause of ADEM.[12] Failure to identify a viral agent suggests that the inciting agents are unusual or cannot be recovered by standard laboratory procedures.[6]

Our patient responded dramatically to early intervention with intravenous methylprednisolone and subsequently underwent successful renal transplantation. Though there have been no well-defined controlled clinical trials, corticosteroids have been considered as the first-line management in the treatment of ADEM. If started early, the long-term prognosis is good in more than 60% cases.[1] In cases where there is failure of corticosteroids, other treatment modalities like plasmapheresis, intravenous immunoglobulins and cytotoxic drugs like cyclosporine have been advised.

In conclusion, an early diagnosis of ADEM and early therapeutic intervention with steroids followed by renal transplantation provided a new lease of life to the child with ESRD.
